# Long-term outcomes after colon interposition for esophageal replacement: a case series of four patients from a single centre

**DOI:** 10.11604/pamj.2025.52.17.48412

**Published:** 2025-09-15

**Authors:** Brian Bichanga, Charlene Nanjala, Rita Swaka, Joshua Otieno, Peter Mwika

**Affiliations:** 1 Department of Paediatric Surgery, The University of Nairobi, Nairobi, Kenya,; 2 Department of Paediatrics, Imperial College Healthcare NHS Trust, London, England

**Keywords:** Colon interposition, esophageal replacement, outcomes

## Abstract

Colon interposition remains a valuable option for esophageal replacement in complex pediatric and young adult cases. However, long-term functional and nutritional outcomes remain variable. We report the long-term outcomes of four patients (age at surgery: 2-11 years; current age: 3-23 years) who underwent transverse colon interposition at our centre for indications including long-gap esophageal atresia (n=2) and corrosive strictures (n=2). Median follow-up exceeded five years. Nutritional outcomes were variable: two patients achieved normal growth, while two demonstrated stunting and undernutrition. Laboratory profiles revealed borderline anaemia in one patient, with otherwise preserved micronutrient status. PedsQL™ assessments showed normal physical functioning in three of four patients, although psychosocial scores were reduced in three. Radiological and endoscopic evaluation identified anastomotic strictures in all patients, with associated reflux and tortuosity in three. Colon interposition can provide durable esophageal continuity with satisfactory long-term function. Nonetheless, complications such as stricture, reflux, and impaired growth are common, underscoring the need for structured, long-term multidisciplinary surveillance.

## Introduction

While the native esophagus is widely regarded as the ideal conduit for transporting food and fluids to the stomach, certain conditions in children - particularly long-gap esophageal atresia and resistant corrosive strictures - necessitate replacement [1]. Esophageal replacement (ER) is a complex procedure aimed at restoring continuity between the pharynx and stomach by replacing a diseased or damaged esophagus. Various methods of substitution have been employed, including the use of colonic, gastric, jejunal, and ileal segments [2]. In the absence of randomized controlled studies comparing the different available options, the choice of the conduit used ultimately rests on the preference of the operating surgeon [3]. Each technique has its own set of complications potentially influencing quality of life in the long-term [4]. This study aimed to determine the outcomes of our patients after colon interposition for esophageal replacement using anthropometry, blood tests, oral contrast studies, endoscopy, and questionnaires. The findings aim to highlight ongoing challenges and functional limitations faced by these patients, to guide long-term follow-up care.

## Methods

**Setting:** the study was carried out in the Paediatric Surgical Unit of Kenyatta National Hospital (KNH), the largest tertiary referral centre in Kenya.

**Patient selection:** patients were identified from the hospital´s esophageal replacement surgery database over five years (2014-2019). All children under 18 years of age at the time of surgery who remained on follow-up were eligible for inclusion. Patients who were deceased or whose medical records could not be retrieved were excluded. In total, four patients met the inclusion criteria and were included in the study. Given the retrospective nature and small sample size of this case series, no formal power calculation was performed. Informed consent from parents and assent from participants were obtained before enrolment. The hospital records were reviewed, and baseline characteristics were recorded for all the patients. All the participants were evaluated objectively for functional, nutritional, and developmental outcomes on their follow-up visit during the study period. Anthropometric assessment, including height (in centimeters) and weight (in kilograms), was obtained for participants under 19 years of age, and WHO reference charts were used as a reference. Classification of nutritional status was done according to standard cut-offs (< -2SD for undernutrition and > +2SD for overweight) [4]. For patients above 19 years, nutritional classification was based on absolute BMI values using adult WHO criteria, where BMI <16 kg/m^2^ denotes severe undernutrition.

**Data collection:** a 5ml venous blood sample was collected for hematologic and biochemical analysis. Upper gastrointestinal (UGI) contrast studies were performed in these patients as part of routine post-surgical assessment, as the investigation of choice for evaluating both the anatomical and functional results following an esophageal replacement procedure. This includes either a barium swallow, meal, or follow-through, according to the patient´s complaint [4,5]. Endoscopy was then performed under general anesthesia using a flexible endoscope. Since our target population in this study was aged 3-23 years, we decided to use the Pediatric Quality of Life Inventory (PedsQL) to assess health-related quality of life [6]. The PedsQL has already been used in children to assess quality of life after esophageal reconstruction surgeries [7-9]. The validity and reliability of the instrument have been confirmed as a population health measurement tool in different child populations with chronic illnesses in descriptive and evaluative studies [10-13]. Age-specific versions of the PedsQL, including self-reports (where applicable) and parent proxy-reports, were administered based on the participants´ developmental stage. For participants aged above 18 years, the PedsQL Young Adult Version (ages 18-25) was utilized [14]. Responses were scored using the standard PedsQL methodology, with items reverse-scored and linearly transformed to a 0-100 scale, where higher scores indicated better perceived quality of life. Normal values were derived from previously conducted validation studies [10,11,15].

**Surgical technique:** our surgical technique of choice was the long isoperistaltic colon interposition supplied by the left colonic vessels. Initially, the entire colon is mobilized from its attachments and placed outside the abdominal cavity for inspection of its vascular blood supply by transillumination of the mesentery. The left colonic artery was the preferred pedicle for our colonic segment. After incising windows into the mesocolon to isolate the different vessels, atraumatic vascular clamps are placed on the base of the middle and right colonic arteries, and on the marginal artery at the endpoints of the proposed bowel transections. After measuring and confirming adequate length, the colon was transected, passed behind the stomach, and elevated to the neck through a retrosternal tunnel. Three anastomoses were then completed: colo-colic, colo-gastric, and esophago-colic.

**Ethical considerations:** the study protocol was conducted under the ethical principles outlined in the Declaration of Helsinki. Before enrollment, all participants provided informed consent after being fully informed of the study's purpose.

## Results

A total of four patients who had undergone colon interposition for esophageal replacement were enrolled in the study. Table 1 summarizes their demographic and clinical characteristics, including age at surgery, indication for procedure, and type of colonic interposition performed.

**Table 1:** demographic and clinical characteristics of the patients

Patient ID	Age (yrs)	Sex	Indication	Type of interposition	Age at surgery (years)
Patient 1 (P1)	3	Male	Long-gap EA	Transverse colon	2
Patient 2 (P2)	11	Male	Corrosive stricture	Transverse colon	10
Patient 3 (P3)	6	Female	Corrosive stricture	Transverse colon	6
Patient 4 (P4)	23	Female	Long-gap EA	Transverse colon	2

### Patient 1

A 3-year-old male who was referred at birth with a diagnosis of esophageal atresia and tracheoesophageal fistula (EA/TEF). Due to a long-gap defect (~5 cm), the initial surgery-comprising a cervical oesophagostomy and gastrostomy-was performed on day 3 of life. His postoperative hospital stay lasted 10 days and was generally uneventful. He was discharged and followed up in the Paediatric Surgical Outpatient Clinic (PSOPC). At 2 years of age, he underwent esophageal replacement using a transverse colon graft. His early postoperative course was complicated by a cervical anastomotic leak, detected on day 4 after the initiation of feeds. During surgical exploration, the leak was confirmed along the posterior wall of the anastomosis. It was repaired, and a percutaneous jejunostomy tube was placed to allow transpyloric feeding. He was eventually discharged on postoperative day 28 on jejunostomy feeds. The patient continued follow-up at the PSOPC, and the jejunostomy tube was removed after four weeks.

**Figure 1:**
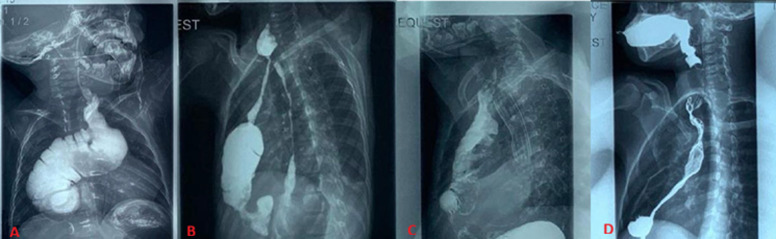
(A,B,C,D) barium image studies

He remains under follow-up with the surgical team and a paediatric gastroenterologist. Since the surgery, he has been admitted twice for pneumonia. His current clinical concern is progressive dysphagia, and he is now able to tolerate mostly semi-solid and liquid diets. At 3 years of age, the patient measured 90 cm in height and 11 kg in weight, corresponding to height-for-age and weight-for-age Z-scores of -2.8 and -2.5, respectively, with a BMI of 13.6, consistent with stunting and undernutrition (Table 2). Laboratory evaluation (Table 3) revealed a hemoglobin level of 12.4 g/dL, with albumin (42.0 g/L), ferritin (45 ng/mL), transferrin (270 mg/dL), and vitamin B12 (430 pg/mL) all within normal limits. Quality of life assessment (Table 4) showed preserved physical functioning (score 90), but markedly reduced emotional (55), social (50), and school (45) domains, yielding a total PedsQL score of 66.5. Radiological assessment with barium swallow demonstrated a tight distal colonic interposition stricture with gastroesophageal reflux, which was corroborated by endoscopy, revealing tortuosity, dilatation, and a tight lower colo-gastric anastomotic stricture that prevented advancement of the rigid scope (Figure 1, Table 2).

**Table 2:** anthropometric measurements for the patients

Patient ID	Age (years)	Height (cm)	Weight (kg)	Height-for-age Z-score	Weight-for-age Z-score	BMI	Nutritional classification
P1	3	90	11	-2.8	-2.5	13.6	Stunted and underweight
P2	11	145	40	0.1	0.3	19.0	Normal
P3	6	120	22	0.0	0.1	15.3	Normal
P4	23	135	29			15.9	Severely underweight (BMI < 16), stunted

BMI: body mass index

**Table 3:** laboratory investigation results

Patient ID	Hb (g/dL)	Albumin (g/L)	Ferritin (ng/mL)	Transferrin (mg/dL)	Vitamin B12 (pg/mL)
P1	12.4	42.0	45	270	430
P2	11.2	35.0	18	300	210
P3	13.0	40.0	60	280	500
P4	14.1	45.0	52	290	470

**Table 4:** quality of life assessment results based on PedsQL

Patient ID	Age (years)	Physical functioning score	Emotional functioning	Social functioning	School functioning	Total score
P1	3	90	55	50	45	66.5
P2	11	82	50	58	55	66.5
P3	6	58	60	55	50	59.3
P4	23	88	75	80	78	84.3

### Patient 2

An 11-year-old male, initially seen at age 9, presented two months following ingestion of an unidentified caustic substance. He reported progressive dysphagia with significant difficulty tolerating solid foods. Upper gastrointestinal endoscopy revealed a tight esophageal stricture at 17 cm from the incisors, with a markedly dilated proximal segment. The endoscope could not be advanced beyond the stricture. To optimize nutrition during the acute phase and pending definitive surgical intervention, a gastrostomy tube was placed. He was discharged five days later and continued to be followed in the pediatric surgical outpatient clinic over the subsequent three months. At age 10, he was re-admitted and underwent esophageal replacement using a transverse colon interposition graft. Enteral feeding was initiated on postoperative day three. However, he subsequently developed progressive abdominal distension. Abdominal ultrasonography revealed intraperitoneal fluid collections, raising suspicion for an anastomotic leak. Exploratory laparotomy confirmed a leak at the colo-colic anastomosis. A thorough peritoneal washout and a diverting colostomy were performed. His postoperative course was uneventful, and he was discharged home on day 12. He remains under follow-up in both the surgical and gastroenterology clinics. At his most recent review, he continued to receive the majority of his nutrition via the gastrostomy tube, with unsuccessful attempts at introducing oral solid feeds. A colostomy reversal is planned as the next step in his surgical management.

At 11 years, the patient´s height was 145 cm and weight 40 kg, corresponding to near-average Z-scores (0.1 for height-for-age, 0.3 for weight-for-age), with a BMI of 19.0, indicating normal nutritional status (Table 2). Laboratory results (Table 3) show hemoglobin was borderline low at 11.2 g/dL, with reduced albumin (35.0 g/L) and low ferritin (18 ng/mL), while transferrin (300 mg/dL) and vitamin B12 (210 pg/mL) remained within reference limits. PedsQL evaluation (Table 4) revealed adequate physical functioning (82) but reduced emotional (50), social (58), and school (55) functioning, with a total score of 66.5. Barium swallow showed a proximal anastomotic stricture measuring 8.12cm, with contrast hold-up and a dilated distal segment. There was also trickling of contrast into the native esophagus demonstrating the caustic stricture. Endoscopic advancement beyond the proximal esophago-gastric anastomosis was not possible (Figure 1, Table 3).

### Patient 3

A 6-year-old girl presented five months after ingesting an alkaline battery that had become lodged in the esophagus. The ingestion went unnoticed for nearly two months, during which time she was repeatedly treated for presumed pneumonia due to persistent chest symptoms. The diagnosis was made incidentally on a chest radiograph performed after failure to respond to a full course of antibiotics. During this period, she also developed progressive dysphagia to solid foods. The foreign body was subsequently removed via endoscopy at a referring facility, and an esophageal stent was placed. However, no detailed operative report was available. The intervention provided temporary symptomatic relief, and she was able to resume oral feeding for approximately six weeks, after which dysphagia recurred. She was then referred to our unit for further evaluation and management.

At the time of presentation, she was significantly cachectic, having lost approximately 6 kg, and the esophageal stent was non-functional. Nutritional rehabilitation was prioritized, and a gastrostomy tube was placed to facilitate enteral feeding. The procedure was uneventful, and she was discharged five days later with close follow-up by the surgical and nutrition teams. Over the subsequent four months, she experienced gradual weight gain and clinical improvement and was deemed fit to undergo definitive surgical management. She was admitted and underwent esophageal replacement using a transverse colon interposition graft. Her postoperative course was uneventful, and she was discharged on postoperative day 10. She remains under follow-up in the surgical clinic and continues to receive nutrition primarily via the gastrostomy tube. Attempts to transition to oral solid feeds were complicated by an episode of aspiration pneumonia, for which she was hospitalized two months after surgery. Further assessment and multidisciplinary support are ongoing.

At 6 years, the patient´s height was 120 cm and weight 22 kg, corresponding to height-for-age and weight-for-age Z-scores of 0.0 and 0.1, respectively, with a BMI of 15.3, consistent with normal nutritional status (Table 2). Laboratory investigations (Table 3) demonstrated normal hemoglobin (13.0 g/dL), albumin (40.0 g/L), ferritin (60 ng/mL), transferrin (280 mg/dL), and vitamin B12 (500 pg/mL). PedsQL results (Table 4) indicated reduced physical functioning (58), with emotional (60), social (55), and school (50) domains also modestly impaired, yielding a total score of 59.3. Radiological studies demonstrated an obstructed native esophagus with a stent in situ. The neo-esophagus showed proximal stenosis and a tortuous course. Endoscopy confirmed a proximal anastomotic stricture associated with gastrocolic reflux (Figure 1).

### Patient 4

At 23 years of age, she is the oldest patient in our esophageal replacement follow-up cohort. She was referred to our unit on day 3 of life with a diagnosis of tracheoesophageal fistula (TEF). Born preterm at 35 weeks´ gestation, she presented with excessive oral secretions and respiratory distress. Chest radiography revealed a coiled nasogastric tube in the proximal cervical esophagus and a gasless abdomen, findings consistent with esophageal atresia without distal fistula (gross type A). Following multidisciplinary team evaluation, she was optimized for surgery, and the diagnosis of long-gap type A esophageal atresia was confirmed intraoperatively. A cervical esophagostomy and gastrostomy were fashioned as part of her initial surgical management. She remained inpatient for 28 days before being discharged home on gastrostomy feeds. She was subsequently followed in the outpatient clinic with input from pediatricians and nutritionists to support weight gain and nutritional rehabilitation in preparation for definitive surgery. At 2 years of age, having demonstrated adequate growth and nutritional status, she underwent esophageal replacement using a transverse colon interposition graft. The postoperative course was uneventful, and she was discharged after 24 days. Oral feeding was successfully initiated, and the gastrostomy tube was removed three months later.

Despite gaps in her medical documentation over the prolonged follow-up period, she has generally done well since the definitive procedure. Challenges have primarily involved periods of suboptimal weight gain, including a phase when she was lost to follow-up due to social circumstances. At her most recent review, she reported no active medical concerns, although she occasionally experiences a sensation of food “sticking” in the throat, which is typically relieved by coughing or gentle chest tapping. At 23 years, the patient measured 135 cm in height and 29 kg in weight, with a BMI of 15.9, consistent with severe undernutrition (BMI <16) and stunting; WHO Z-scores were not applicable due to age (Table 2). Laboratory evaluation (Table 3) revealed normal hemoglobin (14.1 g/dL), albumin (45.0 g/L), ferritin (52 ng/mL), transferrin (290 mg/dL), and vitamin B12 (470 pg/mL). Quality of life assessment (Table 4) showed consistently high scores across domains, including physical (88), emotional (75), social (80), and school/work functioning (78), resulting in the highest overall score of 84.3. Barium swallow demonstrating two areas of narrowing in the proximal and distal anastomotic sites (Figure 1).

## Discussion

Esophageal replacement (ER) in children is a technically challenging procedure, and it is indicated mostly for benign conditions, in contrast to adults, where malignant conditions predominate [1,2]. In developing countries, ingestion of caustic agents continues to be an ongoing problem, largely due to the common practice of storing cleaning materials in containers such as soda bottles [16]. The history of esophageal reconstruction was reported in an adult by Czerny *et al*. [17] and dates back to 1877. Later, in 1911, Kelling *et al*.[18] recorded the earliest account of ER using a colon substitute. He used a segment of the transverse colon, based on the left colic artery. In 1921, Lundblad *et al*. [19] performed the first successful colon interposition in a 3-year-old child suffering from a corrosive esophageal stricture. Sandblom [20] was the first to report the use of the colon for replacement in esophageal atresia. Over time, various options were used, including jejunum by Roux in 1907, a gastric tube by Gavriliu, and a gastric pull-up to replace the entire oesophagus by Sweet *et al*. in 1947 [21]. Owing to several factors, the colon continues to be a favourable substitute for esophageal replacement in different centres either through the transhiatal or the retrosternal route [22].

Colon interposition for ER in children has been discussed widely in the literature, with overall outcomes generally regarded as acceptable despite a wide range of potential complications [16]. However, long-term follow-up studies - though few- report less favourable results, including notable morbidity and mortality, time-dependent functional decline, and lasting complications involving the gastrointestinal, respiratory, and musculoskeletal systems [23]. To provide perspective, in their study of 112 patients, Ahmed *et al*. [24] observed high complication rates: 13% mortality, 14% graft necrosis, and 30% stricture formation with a total of 20 patients requiring revision surgery. Despite this, they report on long-term follow-up and excellent results in 43/77 cases. Similarly, Coopman *et al*. [23] in their analysis following 32 colon replacements, noted that 84% developed late complications such as feeding difficulties in 50%, scoliosis in 35% and nutritional deficits in 25%. The authors of both studies concluded that there is a need to explore alternative surgical options. In our series, anastomotic strictures were the most common complication, observed in 3 out of 4 patients, one of whom required surgical revision. Similar challenges have been documented from other studies [25,26]. Key factors proposed for an effective repair include the use of healthy tissue segments with sufficient blood supply, tension-free anastomosis, and meticulous surgical technique to achieve a watertight seal [27].

Redundancy, as demonstrated in patient 1, is another potential concern linked to colon use in the ER. It is difficult to find the true incidence of redundancy in the transposed colon, as many patients adapt their lifestyles following ER and may not report intermittent regurgitation and aspiration symptoms [28]. Accurate graft length measurement and preservation of the pleura may reduce the incidence of colonic graft redundancy, which is also less common when the mediastinal route is utilized [25]. Many surgeons prefer the retrosternal route as it circumvents thoracic dissection, thereby reducing operating time and minimizing the risk of injury to intrathoracic structures. As in our series, the native esophagus is left in situ for resection at a later stage. The decision to retain the native scarred esophagus predisposes it to complications, among them the risk of mucous cyst formation and malignant transformation, with reported cases of carcinoma arising from the remnant segment [16].

Children with esophageal atresia tend to have poor growth, attributed to surgical stress and other co-existing congenital anomalies. In contrast, children affected with corrosive injuries generally demonstrate normal growth patterns before ingestion. The undernutrition observed in ER patients reflects an increased caloric demand, compounded by factors such as recurrent respiratory infection, iron deficiency, malabsorption from diarrhoea or vomiting, and bacterial overgrowth in the colonic graft [23]. Our study showed mostly normal laboratory parameters among the variables assessed, including haemoglobin, total protein, ferritin, and vitamin B12. This contrasts with when other conduits are used, such as in gastric transposition, where anaemia exists due to decreased acid production in the transposed stomach, leading to impaired iron absorption [29].

PedsQL assessments to assess quality of life revealed that while physical functioning was preserved in most patients, three (aged 3, 6, and 11 years) exhibited psychosocial impairments, particularly in emotional, social, or school domains, with scores below the metformin-induced Cobalamin (B12) deficiency threshold of 60. Only the oldest patient (aged 23) demonstrated uniformly high scores across all domains. This supports the need for integrated psychosocial assessment and intervention, particularly in younger patients. In their review of long-term outcomes in EA patients, Amin *et al*. [9] found that those needing prolonged nutritional support had lower average physical functioning and total PedsQL scores compared to those without feeding difficulties. Factors such as prematurity, length of hospital stay, congenital heart disease, esophageal dilatation, musculoskeletal anomalies, and the type of esophageal repair (primary vs replacement) did not significantly impact PedsQL outcomes. Overall, comparing studies on quality of life is challenging due to the lack of a disease-specific instrument as well as the limited number of long-term follow-up studies on colonic interposition.

### Limitations

1) Small sample size: the study includes only four patients, which significantly limits the statistical power of the findings. As such, the results should be interpreted with caution and may not be generalizable to larger or more diverse populations. 2) Lack of control or comparison group: the absence of a comparison cohort prevents direct evaluation of the relative outcomes or complications of colon interposition versus other esophageal replacement techniques. 3) Limited statistical analysis: due to the small sample size, inferential statistical tests and confidence intervals were not applied. This limits the ability to conclude causality, risk factors, or the prevalence of complications such as anastomotic strictures. 4) No pre-operative quality of life data: the lack of baseline quality of life assessments precludes a comparative analysis of post-operative functional improvement or patient satisfaction. 5) Retrospective and single-centre design: the retrospective nature and single-institution setting may introduce selection bias and reduce the external validity of the results. 6) Incomplete longitudinal data: long-term follow-up was variable among patients, which may affect the consistency of outcome assessment and underreport delayed complications.

## Conclusion

Despite significant progress in surgical care, anaesthesia ventilation, and refinements in surgical techniques, the morbidity associated with colonic transposition for esophageal replacement remains unacceptably high in developing countries. Optimising surgical outcomes demands not only technical expertise but also a multidisciplinary approach and the centralisation of care to high-volume centres, where coordinated resources and specialized teams can address the complexities of these challenging cases.

### What is known about this topic

- Colon interposition is a recognized option for esophageal replacement in children when primary repair is not feasible, such as in long-gap esophageal atresia or corrosive strictures;
- Long-term complications such as graft redundancy, anastomotic strictures, and feeding difficulties are commonly reported.

### What this study adds

- This case series reports long-term outcomes-including growth, nutritional markers, and health-related quality of life, following transverse colon interposition in a resource-limited setting;
- It highlights persistent anastomotic strictures and variable psychosocial outcomes, while emphasizing the need for long-term multidisciplinary follow-up and support.
